# Clinical audit of core podiatry treatment in the NHS

**DOI:** 10.1186/1757-1146-2-7

**Published:** 2009-03-13

**Authors:** Lisa Farndon, Andrew Barnes, Keith Littlewood, Justine Harle, Craig Beecroft, Jaclyn Burnside, Tracey Wheeler, Selwyn Morris, Stephen J Walters

**Affiliations:** 1 Podiatry Services, Sheffield PCT, Jordanthorpe Health Centre, Sheffield, S8 8DJ, UK; 2 Podiatry Department, Barnsley PCT NHS Trust, New Street Clinic, Upper New Street, Barnsley, S70 1LP, UK; 3 Podiatry Department, North Lincolnshire & Goole Hospital NHS Foundation Trust, Diana Princess of Wales Hospital, Scartho Road, Grimsby, DN33 2BA, UK; 4 Department of Foothealth, Bassetlaw PCT, Retford Hospital, North Road, Retford, Notts, DN22 7XF, UK; 5 Podiatry Department, Ashfield Health Village, Kirkby-in-Ashfield, Notts, NG17 7AE, UK; 6 Podiatry Department, Nottinghamshire County Teaching PCT, Park House Health and Social Care Centre, 61 Burton Road, Carlton, Nottingham, NG4 3DQ, UK; 7 Podiatry Services, Doncaster PCT, East Laith Gate House, East Laith Gate, Doncaster, DN1 1JE, UK; 8 Lincolnshire PCT, Podiatry Department Marisco Medical, Stanley Avenue, Mablethorpe, LN12 1DP, UK; 9 ScHARR, University of Sheffield, Regent Court, 30 Regent St, Sheffield, S1 4DA, UK

## Abstract

**Background:**

Core podiatry involves treatment of the nails, corns and callus and also giving footwear and foot health advice. Though it is an integral part of current podiatric practice little evidence is available to support its efficacy in terms of research and audit data. This information is important in order to support the current NHS commissioning process where services are expected to provide data on standards including outcomes. This study aimed to increase the evidence base for this area of practice by conducting a multi-centre audit in 8 NHS podiatry departments over a 1-year period.

**Methods:**

The outcome measure used in this audit was the Podiatry Health Questionnaire which is a self completed short measure of foot health including a pain visual analogue scale and a section for the podiatrist to rate an individual's foot health based on their podiatric problems. The patient questionnaire was completed by individuals prior to receiving podiatry care and then 2 weeks after treatment to assess the effect of core podiatry in terms of pain and foot health.

**Results:**

1047 patients completed both questionnaires, with an age range from 26–95 years and a mean age of 72.9 years. The podiatrists clinical rating at baseline showed 75% of patients had either slight or moderate podiatric problems. The differences in questionnaire and visual analogue scores before and after treatment were determined according to three categories – *better, same, worse *and 75% of patients' scores either remained the same or improved after core podiatry treatment. A student t-test showed a statistical significant difference in pre and post treatment scores where P < 0.001, though the confidence interval indicated that the improvement was relatively small.

**Conclusion:**

Core podiatry has been shown to sustain or improve foot health and pain in 75% of the patients taking part in the audit. Simple outcome measures including pain scales should be used routinely in podiatric practice to assess the affect of different aspects of treatments and improve the evidence base for podiatry.

## Background

A large number of the general population experience foot problems which is highlighted by a review of foot survey data from the UK and overseas (Australia, America and Europe) [1]. Various methods have been used to survey the incidence of foot problems, including an examination by a professional, face to face or telephone interviews and postal questionnaires. A summary of combined surveys found that between 20–78% of people suffer from corns, callus and bunions, between 20–49% have lesser toe deformities and 28–56% have toenail problems [1]. The incidence and types of foot problems are variable when reported via surveys due to the types of populations studied and whether foot problems are self reported or assessed by a health professional. In the past the vast majority of surveys have concentrated on foot problems in older people, whether in residential care, on a hospital ward or living in the community. Understandably, when a professional diagnoses and reports foot problems the incidence is higher than when compared with those that are self reported. The high incidence of foot pathologies in the population is reflected in the number of people accessing podiatry care; the most recent figures available show 2 million people are treated annually by the NHS, 769,000 of these are new episodes of care of which 56% are for older people [2].

Podiatry practice includes the treatment of foot pathologies associated with the nails and soft tissues, such as corns and callus; which is regarded as core podiatry treatment and is required for these types of conditions [3]. Currently, little evidence exists to support the efficacy of core podiatry treatments though anecdotally podiatrists believe them to be beneficial. Some studies have investigated the pain relieving properties of scalpel debridement. One multi-centre NHS based project included 79 patients and found that they reported a reduction in pain after treatment when pre and post operative pain scores using a Visual Analogue Scale (VAS) were used and this was statistically significant, though the benefit was not sustained [4]. Reduction of callus with a scalpel was also found to reduce pain again using a pain VAS directly after treatment and seven days later, in conjunction with improving functional ability in a small group of older adults [5]. A small qualitative study using semi-structured interviews with older people, found that core podiatry treatment gave both a physical benefit to those who receive it as well as some degree of emotional reassurance as having continued care was felt to sustain individuals' foot health [6]. Current research priorities identified for podiatric practice also include *treatment effectiveness*, as a major issue requiring further investigation [7], and one way to explore this is to use an outcome measure.

In the last decade, specific podiatric outcome measures have been developed to measure the efficacy of different types of interventions and treatments.

The Foot Function Index (FFI) was designed and validated in a study by Budiman-Mak and colleagues [8] to assess in terms of pain, disability and activity restriction; the impact that foot pathologies have on function. Bennet and Patterson [9] describe the development of The Foot Health Status Questionnaire (FHSQ), which is designed to measure foot health related quality of life. Other measures have been developed which are more patient centred. Garrow et al [10] developed and validated a tool to measure foot pain and disability sensitive to individuals with a range of different problems affecting mobility. Waxman and colleagues [11] later used this in a randomised controlled trial measuring the effect of a self-care foot programme for older people. The Bristol Foot Score [12] was formulated after consultations with groups of patients and individuals. The authors suggest that patient as well as practitioner views should be considered when assessing the usefulness and efficacy of different podiatric interventions.

The Podiatry Health Questionnaire (PHQ) was developed to be self completed by patients and was evaluated by Macran et al [13] in 2038 individuals across four UK podiatry departments. It consists of 6 foot related questions with a choice of 3 responses for each around the dimensions of walking, foot hygiene, nail care, foot pain, worry about feet and impact on quality of life. It was used in combination with a visual analogue pain scale (VAS) and a Podiatry Objective Clinical Score (POCS) which is clinical measure of current foot problems as determined by a podiatrist and rated from 1 (no problems) to 5 (gross problems) (Additional file 1). The results were compared with a generic measure of health status (EQ-5D) to assess podiatry outcomes. The PHQ was found to be a useful measure of foot health and showed a good correlation between self-reported morbidity in this tool and the EQ-5D.

This paper describes a multi-centre audit of podiatry patients receiving core podiatry care using the PHQ as described by Macran et al [13] to investigate outcomes. The 6 items on PHQ were combined to generate a single score ranging from 6 to 18, with a higher score indicating more severe problems. The 11 point VAS scale ranged from 0 (no pain) to 10 (worst pain).

As changes are being made in the way NHS services are commissioned and delivered [14] it is envisaged that the results of this audit will be able to contribute towards offering evidence for the effectiveness of core podiatric practice.

## Method

As this was a simple audit, ethical committee approval was not required, but all participating patients were asked to give their verbal consent after a full explanation of the audit process was outlined, prior to the questionnaires being completed. Eight podiatry departments in the Trent and South Yorkshire region took part in the audit ranging from a small department consisting of 4 staff and serving a mainly rural population to one with over 50 staff with a mixed rural and urban population. A random sample of adult patients who attended core community podiatry clinics in the 8 regions were asked to complete the PHQ prior to their treatment. The podiatrist carrying out the treatment then completed the podiatry objective clinical score, which categorises a patient into one of five sections according to the severity of their foot problems (5 representing gross problems). All podiatrists taking part were given brief training in how to complete the questionnaire, but inter-rater reliability tests were not conducted, as the study was trying to replicate current practice. After each treatment had concluded, a follow up PHQ was given out to each patient and they were asked to complete and return it 2 weeks after their initial treatment. This was decided as the most appropriate time frame for a treatment outcome to be measured, as it was thought that asking the patient to complete the questionnaire directly after treatment might be introducing an element of bias, as the patient may feel obliged to give a favourable opinion. The date when the second questionnaire should be completed was written on the form to remind the patient when it should be filled in.

Inclusion criteria for the audit were; patients who were attending core podiatry treatment, who were able to give verbal consent and were over 18 years old. Data was collected over a 12-month period in each of the 8 podiatry departments. Departments then entered their data onto an Excel spreadsheet and results were combined and migrated into the Statistical Package for Social Scientists (SPSS) for statistical analysis. The PHQ-score and VAS outcomes were regarded as continuous outcomes. The change in PHQ-score and VAS from baseline to 2 weeks was compared using a paired t-test. A 95% confidence interval (CI) for the mean change in scores over time was also calculated. A p-value of < 0.05 was regarded as statistically significant. The change in VAS and PHQ score from baseline to 2 weeks was also categorised into three levels; *same*, *better *or *worse*, with a *same *category corresponding a change score of 0.

## Results

### Baseline data (see Table 1 &2)

**Table 1:** Baseline demographics of audit patients (n = 1047)

	**n**	**mean**	**standard deviation**	**minimum**	**maximum**
**Age**	1047	72.9	11.1	26	95
**VAS**	1019	4.8	2.8	0	10
**Questionnaire score**	1047	11.8	6	6	18
**Podiatry clinical score**	1042	2.8	1	1	5

*For the VAS, questionnaire and Podiatry Clinical Scores; a higher number represents more severe problems

**Table 2:** Baseline demographics of audit patients (cont)

		**n**	**%**
**Location**	**Podiatry Service 1**	109	10.4
	**Podiatry Service 2**	76	7.3
	**Podiatry Service 3**	101	9.6
	**Podiatry Service 4**	25	2.4
	**Podiatry Service 5**	49	4.7
	**Podiatry Service 6**	126	12
	**Podiatry Service 7**	14	1.3
	**Podiatry Service 8**	547	52.2
**Gender**	**Male**	382	36.5
	**Female**	664	63.5
**Patient Status**	**Current Patients**	946	90.4
	**New Patients**	100	9.6
	**Diabetes**	325	31.7
**Podiatry objective clinical score (POCS)**	**No problems**	57	5.5
	**Slight problems**	357	34.3
	**Moderate problems**	427	41
	**Severe problems**	181	17.4
	**Gross problems**	20	1.9

One thousand and forty-seven patients receiving core treatment completed and returned both questionnaires. The response rate was not calculated as not all services kept a tally of the number of questionnaires originally given out.

Tables 1 and 2 show the baseline clinical and demographic characteristics of the patients. The mean age was 72.9 years (range 26–95 years) with 63.5% females and 36.5% males. Ninety per cent (946) were current patients; the remaining 10% (100) were new patients. Thirty-two per cent (325) of patients had diabetes. The mean pain VAS was 4.8 with 28 missing scores. The questionnaire – How are your feet today? could elicit possible scores between 6 (low need) up to 18 (high need). The mean score for this at baseline was 11.8.

The Podiatry Objective Clinical Score (POCS) indicated that 75% of patients were suffering from slight or moderate podiatric problems, however 5.5% were classed as having no problems, so would probably be attending for nail care or foot care advice only. Table 2 indicates the baseline figures for the 8 centres submitting data.

Table 3 shows the VAS and PHQ scores before and after treatment (59 missing VAS scores). For both outcomes there was a statistically significant improvement in scores after treatment (p < 0.001). However, the 95% confidence intervals for the mean change in scores are relatively small; a change of 0.7 for the VAS scores and 0.6 for the PHQ scores.

**Table 3:** Change in questionnaire and VAS score before and after treatment

		Before		After		Mean	95% CI		
	N	Mean	SD	Mean	SD	Change	Lower	Upper	P-value
Q Score	1047	11.8	2.6	11.2	2.7	0.5	0.4	0.7	< 0.0001
VAS	988	4.8	2.8	4.1	2.7	0.7	0.6	0.9	< 0.0001

*For the VAS, Questionnaire Scores; a higher number represents more severe problems.

A positive mean P-value from paired t-test change implies an improvement or reduction in severity of problems over time.

**Figure 1:**
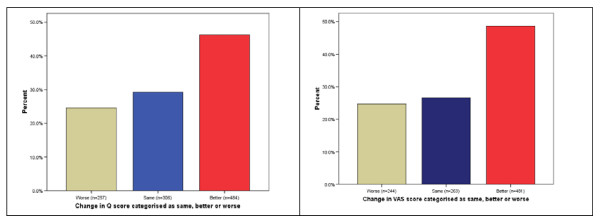
**Changes in questionnaire and VAS scores categorised as worse, same or better**.

The changes in PHQ questionnaire and VAS scores before and after treatment were also reclassified into three categories – *better, same, worse *(see Figure 1). Seventy-five per cent of patients reported that their foot health and pain levels had improved or remained the same after receiving core podiatry treatment, though the remaining 25% stated that they were worse.

## Discussion

Core podiatry treatment was found to give a statistically significant improvement in terms of pain and foot health, though the change was relatively small. Twenty-five per cent of patients reported higher questionnaire scores and pain scores after receiving treatment suggesting that their foot health was deteriorating. This may have occurred for a number of reasons. The average age of this group was 72 years, and older people may possibly be suffering from complex medical problems that could affect their pain and mobility [15]. Macran et al's study which first used the PHQ found that from a sample of 2073 patients with the same mean age of 72 years, 82% were being treated for one of the following conditions – rheumatism/arthritis, respiratory problems, heart/circulatory disorders, diabetes and cancer.

The first two statements of the PHQ are about walking and hygiene and therefore a general measure of foot health which may not be altered after receiving podiatry treatment. Some patients may have been worried that they could be discharged if they reported greatly improved foot health as most NHS podiatry services now have access and discharge criteria based on clinical need. Some podiatrists taking part in the audit reported that some patients did not really understand how to fill in the pain VAS, which again may account for some higher than expected scores and the missing data. If this audit was repeated, help should be offered and further explanation if necessary to patients when they are completing the VAS to ensure that it is completed in the correct manner.

The time scale to complete the post treatment questionnaire for core podiatry was decided at 2 weeks. If the questionnaire had been completed straight after a core treatment, this again may have given improved outcome scores as the greatest benefit may be felt at this time. However, the optimum time to achieve the maximum advantage from a podiatry treatment in terms of foot health and pain has never really been determined, though a two week follow up has been used before in a similar audit [12]. The majority of patients (over 70%) reported an improvement or no change in their foot health and pain scores after treatment. In a group receiving core podiatry care, this might be expected, as sustaining foot health is an acceptable outcome in people who may have mobility problems and pain, some of which may be associated with systemic diseases. This concurs with the results of another study which used the Bristol Foot Score as an outcome measure and found no statistical improvement in foot health 2 weeks after core podiatry treatment in a group with a similar mean age [12]. However, withdrawing podiatry care in older people classed as 'low risk' has been found to lead to the development of more serious foot problems in some and this is associated with a reduction in independence and ageing [15]. This group of people are also more likely to have mobility problems, which can result in difficulties providing self foot care [16].

The outcome measure used consisted of some general questions to give an indication of self-care ability, which is useful when assessing need for core podiatry care. The self-assessment questionnaire did not really take into account that some patients may have been suffering from co-morbidities which may have been affecting their pain and foot health and that might not be improved by podiatry care. Campbell [17] recommends that a universal assessment tool is required in podiatric practice to measure foot health in older low risk people. She suggests this should include assessments of foot health, self-care, callused feet, neurological and vascular problems. Such a tool could be developed with different domains associated with the different specialities in current podiatry and be applicable to all ages.

## Conclusion

In this large multi-centre audit core podiatry treatment was shown to improve outcomes in terms of foot health and pain which was statistically significant. The improvements however were relatively small which highlights that the podiatry profession needs to determine what a clinically significant improvement is before further work in this area can be carried out to assess the effectiveness of core podiatry care or other aspects of podiatric practice.

It is important for simple outcome measures to be incorporated into day-to-day clinical care to ensure that ongoing treatments are evaluated and evidence is available to support such interventions. The VAS pain scale was a relatively simple tool to use and could easily be incorporated into current patient records including paper and electronic systems, though some older people required some help with its completion. Those with visual problems would be disadvantaged, but a verbal description made to the clinician could be substituted if required.

As the sample sizes differed dramatically in the departments taking part in this audit, it is difficult to adequately benchmark clinical outcomes across the region. But the overall sample size, though not representing the total population of those receiving core podiatry care, is still a large number of patients, compared with most previous reports using outcome measures.

This audit has given some valuable information regarding the effect of core podiatry treatment and has highlighted the need for outcome measures to be incorporated into daily podiatric practice to increase its evidence base.

## Competing interests

The authors declare that they have no competing interests.

## Authors' contributions

LF conceived of the study and drafted the manuscript with the participation of AB, KL, JH, CB, JB, TW, SM and SW. AB collated the results and prepared them for statistical analysis. SW performed the statistical analysis. All authors read and approved the final manuscript.

## Supplementary Material

Additional file 1 **The patient health questionnaire.** Patient questionnaire. Click here for file
