# Supplementary material for: Clinical audit of core podiatry treatment in the NHS
Source: J Foot Ankle Res. 2009 Mar 13;2:7. doi: 10.1186/1757-1146-2-7 (PMC2663778; doi:10.1186/1757-1146-2-7)
Supplement: Additional file 1 — The patient health questionnaire. Patient questionnaire. [file 1757-1146-2-7-S1.doc]

Additional file 1: The patient health questionnaire

**Patient questionnaire: How are your feet today?**

**For each of the 6 questions please tick the statement that best describes how your feet are today?**

**Walking/Getting about**

How much do your feet affect you walking about?

I have no problem in walking about □

I have some problem in walking about □

I have severe problems in walking about □

**Hygiene**

How much of a problem is it for you to wash and dry your feet?

I have no problem washing or drying my feet □

I have some problems washing and drying my feet □

I cannot wash or dry my feet □

**Nail Care**

How much of a problem is it for you to cut or file your own toe nails?

I have no problems cutting/filing my own toe nails □

I have some problems cutting/filing my own toe nails □

I cannot cut/file my own toe nails □

**Foot pain**

Do your feet cause your pain or discomfort?

My feet cause me no pain or discomfort □

My feet cause me some pain or discomfort □

My feet cause me severe pain or discomfort □

**Worry/Concern**

How much are you concerned about the condition of your feet?

I am not concerned about the condition of my feet □

I am somewhat concerned about the condition of my feet □

I am greatly concerned about the condition of my feet □

**Quality of life**

Do your foot problems affect the quality of your life?

My foot problems do not affect the quality of my life □

My foot problems have some effect on the quality of my life □

My foot problems have a severe effect on the quality of my life □


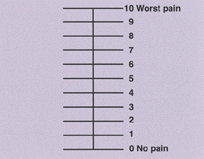


Please draw a line at the point which best describes how good or bad your feet are today

**Thank you for completing this, can you now please give this to the podiatrist.**

**Appendix 1 (cont)**

**Podiatry objective clinical score (POCS)**

This is an objective score given by the podiatrist. It is designed to reflect your judgement of the patient’s foot health. To achieve standardisation of scoring from 1 (best foot health) to 5 (worst foot health) please follow the guidelines below:

**POCS – 1 NO PROBLEMS**

Asymptomatic foot

No nail/skin pathologies

**POCS 2 – SLIGHT PROBLEMS**

Pathological nails, slight thickening and/or lightly involuted nails

Light callus and/or small corn(s)

**POCS 3 – MODERATE PROBLEMS**

Pathological nails, moderate thickening and/or moderately involuted nails

Extended areas of callus and/or corn(s)

Small necrotic lesion

Minor functional abnormality – valgus, varus, HV, heel pain, metatarsalgia

Foot strain

Verrucae

**POCS 4 – SEVERE PROBLEMS**

Patholgical nails, severe thickening or ingrowing nails with tissue breakdown

Moderate to severe functional deformity – equinus etc

Larger areas of callus and/or several corns

Larger necrotic lesion and/or ulceration

**POCS 5 – GROSS PROBLEMS**

Gross functional deformity

Gangrenous tissue present

Generalised bacterial infection

Septicaemia

Osteomyelitis

Malignancy

**POCS score** □
